# Do Nutritional Factors Interact with Chronic Musculoskeletal Pain? A Systematic Review

**DOI:** 10.3390/jcm9030702

**Published:** 2020-03-05

**Authors:** Ömer Elma, Sevilay Tümkaya Yilmaz, Tom Deliens, Iris Coppieters, Peter Clarys, Jo Nijs, Anneleen Malfliet

**Affiliations:** 1 Pain in Motion International Research Group, Department of Physiotherapy, Human Physiology and Anatomy, Faculty of Physical Education & Physiotherapy, Vrije Universiteit Brussel, 1090 Brussels, Belgium; omerelma@vub.ac.be (Ö.E.); sevilay.tumkaya.yilmaz@vub.be (S.T.Y.); iris.coppieters@vub.be (I.C.); jo.nijs@vub.be (J.N.); 2 Department of Physiotherapy, Human Physiology and Anatomy, Faculty of Physical Education & Physiotherapy, Vrije Universiteit Brussel, 1090 Brussels, Belgium; tom.deliens@vub.be; 3 Department of Movement and Sport Sciences, Faculty of Physical Education and Physiotherapy, Vrije Universiteit Brussel, 1050 Brussels, Belgium; peter.clarys@vub.be; 4 Department of Rehabilitation Sciences and Physiotherapy, Faculty of Medicine and Health Sciences, Ghent University, 9000 Ghent, Belgium; 5 Department of Physical Medicine and Physiotherapy, University Hospital Brussels, 1090 Brussels, Belgium

**Keywords:** chronic pain, musculoskeletal pain, nutrition, diet, dietary pattern

## Abstract

Dietary patterns may play an important role in musculoskeletal well-being. However, the link between dietary patterns, the components of patients’ diet, and chronic musculoskeletal pain remains unclear. Therefore, the purpose of this review was to systematically review the literature on the link between dietary patterns, the components of patients’ diet and chronic musculoskeletal pain. This review was conducted following the “Preferred Reporting Items for Systematic reviews and Meta-Analyses” (PRISMA) guidelines and was registered in PROSPERO with the registration number CRD42018110782. PubMed, Web of Science, and Embase online databases were searched. After screening titles and abstracts of 20,316 articles and full texts of 347 articles, 12 eligible articles were included in this review, consisting of nine experimental and three observational studies. Seven out of nine experimental studies reported a pain-relieving effect of dietary changes. Additionally, protein, fat, and sugar intake were found to be associated with pain intensity and pain threshold. In conclusion, plant-based diets might have pain relieving effects on chronic musculoskeletal pain. Patients with chronic rheumatoid arthritis pain can show inadequate intake of calcium, folate, zinc, magnesium, and vitamin B6, whilst patients with fibromyalgia can show a lower intake of carbohydrates, proteins, lipids, vitamin A-E-K, folate, selenium, and zinc. Chronic pain severity also shows a positive relation with fat and sugar intake in osteoarthritis, and pain threshold shows a positive association with protein intake in fibromyalgia.

## 1. Introduction

In recent decades, understanding of chronic (musculoskeletal) pain has evolved from a biomedical perspective to a broader approach [1]. It has become evident that chronic musculoskeletal pain has a significant bi-directional relation with various psychological, cognitive, and social factors such as pain catastrophising, kinesiophobia, depression, and anxiety [2,3,4]. Recently, lifestyle factors such as poor sleep, smoking, stress, unhealthy diet, and obesity/overweight, are gaining more attention for chronic pain management [5,6,7,8]. It has been proposed that one or a combination of such intrinsic and extrinsic factors can change the neuronal organisation in the peripheral and central nervous system, leading towards increased sensitivity of the central nervous system (i.e., central sensitization) in musculoskeletal pain conditions such as non-specific chronic lower back pain (NCLBP), osteoarthritis, fibromyalgia, and chronic fatigue syndrome [9,10,11,12,13,14]. Because these influencing factors are highly individual, it becomes obvious that the experience of persistent pain differs from person to person even under the same biological and pathological conditions. Thus, in order to deliver more comprehensive healthcare, these factors should be considered as part of diagnosing and delivering individually tailored multimodal treatment.

The World Health Organisation (WHO) also acknowledges the importance of diet, “Nutrition is coming to the fore as a major modifiable determinant of chronic disease, with scientific evidence increasingly supporting the view that alterations in diets have strong effects (both positive and negative) on health throughout life” [15]. In general, nutrition is an essential part of musculoskeletal health. It has a supportive effect in bone, as well as in cartilage structure and immune modulation [16]. For instance, endogenous pain-relieving systems in the central nervous system require essential fatty acids such as eicosapentaenoic acids, arachidonic acids, and tryptophan [17,18]. These essential fatty acids cannot be synthesized by mammals and need to be derived from food intake. Additionally, vitamin D and calcium intake have been reported as an essential factor for bone health [19], and effects of dietary protein intake on muscle and bone health have been established as well [20]. However, although it is well known that nutrition is an essential part of general musculoskeletal health, it is uncertain how nutritional factors interact with chronic musculoskeletal pain.

Dietary behaviour and dietary intake are lifestyle factors that might influence the occurrence, maintenance, and perception of chronic musculoskeletal pain [21,22,23]. For instance, overweight and obesity are suggested as two main associated factors of unhealthy dietary behaviour and are two main aspects of nutritional status in patients with chronic musculoskeletal pain [24]. Overweight and obesity often occur due to nutrition related underlying mechanisms and are both common comorbidities of chronic musculoskeletal pain disorders such as fibromyalgia [25,26], osteoarthritis [27], chronic lower back pain (CLBP) [28], carpal tunnel syndrome [29], and pelvic pain [30]. They are suggested as risk factors for developing chronic musculoskeletal pain [6]. Prevalence of having chronic musculoskeletal pain increases as body mass index (BMI) raises [6]. Pain severity can show direct relation with overweight and obesity in patients with osteoarthritis [31], lower back pain [32], and fibromyalgia [33,34]. Thus, dietary behavioural change in overweight and obese patients is suggested as an important aspect of pain management [26].

Furthermore, there are some studies that investigate the link between specific nutrients and chronic pain. For instance, it has been reported that chronic lower back pain is associated with low vitamin D intake [35]. Additionally, pain relieving effects of alkaline mineral [36] and omega-3 polyunsaturated fatty acid [37] supplementation have been reported in patients with lower back pain and rheumatoid arthritis, respectively. Moreover, inadequate intake of selenium was found to be associated with pain severity in patients with fibromyalgia, and antioxidant intake was suggested for pain reduction in the same population [38]. However, besides focusing on the effects of single nutrient intake on a specific condition, the consideration of the overall dietary intakes of individuals is suggested as well.

Research on applied diets and dietary patterns considers a dietary evaluation in which overall nutrients and food are examined instead of evaluating a single nutrient or food. This is a natural approach, given that in a diet, nutrients and foods eaten together do not act in isolation, but rather show synergistic effects [39]. Thus, dietary patterns and overall diet analysis are more robust way of understanding diet–disease relationship [40,41]. For instance, assessment of dietary patterns is suggested as an important indicator of chronic low-level systemic inflammation, which is an associated factor of chronic diseases [42]. Adherence to a healthy dietary pattern, such as the Mediterranean diet, is inversely associated with blood inflammatory biomarker levels [43,44]. An anti-inflammatory diet with a lower dietary inflammatory index score relates to a higher (i.e., healthier) score on the Healthy Eating Index–2010 [45,46]. Thus, dietary patterns and overall diet is suggested as an effective therapeutic target for chronic diseases [42].

In sum, diet and dietary patterns are important factors of musculoskeletal health and are suggested as associated factors of musculoskeletal pain. However, it is uncertain how the dietary patterns and components of patients’ diet are associated and interact with chronic musculoskeletal pain. Moreover, to date, there is no clear literature overview bringing together the existing evidence on the association between dietary patterns, components of patients’ diet, and chronic musculoskeletal pain. This overview could give indications of how to include dietary advice in clinical practice for the management of chronic musculoskeletal pain. Therefore, the purpose of this study is to systematically review the existing literature on the link between nutrition and chronic musculoskeletal pain by specifically focusing on dietary patterns, the components of patients’ diet, and pain in patients diagnosed with chronic musculoskeletal pain.

## 2. Methods

### 2.1. Protocol and Registration

This systematic review was conducted and reported in line with the “Preferred Reporting Items for Systematic reviews and Meta-Analyses” (PRISMA) guidelines [47]. The review protocol was registered in the PROSPERO database (https://www.crd.york.ac.uk/prospero/) with the registration number CRD42018110782. 

### 2.2. Search Strategy and Eligibility Criteria

The search strategy was based on the Patient, Exposure, Comparison, Outcome (PECO) framework (P = people with chronic musculoskeletal pain; E = (behavioural) nutrition; C = non-comparison or comparison with a healthy, pain-free population; O = chronic musculoskeletal pain-related outcomes).

Inclusion Criteria

- Studies including adult human volunteers (above 18 years old);
- Having at least 3 months of musculoskeletal pain or musculoskeletal system-originated chronic pain (i.e., chronic musculoskeletal pain);
- Studies that solely investigate the link between nutrition and pain, and do not include any other treatment modality in experimental studies;
- Studies written in English and published in internationally peer-reviewed journals;
- Study types considered were randomised controlled trials, clinical trials (all phases), cohort studies, case–control studies, and cross-sectional studies.

### 2.3. Information Sources and Keywords

The literature search conducted searches using three online databases: PubMed, Web of Science, and Embase by two reviewers (O.E. and S.T.Y.) independently up to 1 October 2018.

Two groups of keywords were combined: “Chronic Musculoskeletal Pain (Patients)” and “Nutrition (Exposure)”. Search terms used in PubMed, Web of Science, and Embase are stated in the Supplementary Materials Section.

### 2.4. Study Selection

Study selection was performed independently by the same two researchers who were blinded to each other (O.E. and S.T.Y.) on the basis of two screening phases. Firstly, titles and abstracts of the search results were checked. Secondly, full texts of possibly relevant articles were checked. Additionally, backward and forward tracking was performed as part of the search strategy. Backward tracking includes screening of the reference lists of relevant articles (i.e., all included studies in the present systematic review as well as other relevant systematic reviews), whereas forward tracking includes searching for studies that cited the studies that were included in the present systematic review. A consensus meeting was organized between the two researchers after every step in the study selection process, where discrepancies between selected studies were discussed. In case of doubts or disagreements between researchers, a third and fourth researcher (A.M. and T.D.) were consulted to make a final decision.

### 2.5. Data Extraction

The following information was extracted from each included paper by two reviewers (O.E. and S.T.Y.) who were blinded to each other: (1) authors, (2) specific chronic musculoskeletal pain condition, (3) study design and duration, (4) participant information, (5) intervention or case group, (6) control group, (7) outcome measures, and (8) findings. All details are listed in Table 1.

### 2.6. Deviation from the Protocol

With reference to the protocol registered in PROSPERO (https://www.crd.york.ac.uk/prospero/) [48] with the registration number CRD42018110782, this systematic review aimed to focus on nutrition as a general aspect. Therefore, the scope of the search included all aspects of the nutrition research such as macronutrients, micronutrients, foods, food groups, dietary supplements, diet analysis, dietary patterns, applied diets, dietary behaviour, etc. Yet, this focus resulted in 149 eligible articles with evidence on the link between nutrition (dietary patterns, dietary supplements, specific nutrients, intravenous nutrient therapy, intramuscular vitamin injection, etc.) and chronic musculoskeletal pain.

However, a review including that many papers with a broad nutritional focus would decrease readability and comprehensibility. Additionally, besides focusing on the effects of single nutrient intake on a specific condition, we aimed to consider overall dietary intakes of individuals as well. Research on dietary patterns considers a dietary evaluation in which overall nutrients and foods are examined instead of evaluating a single nutrient or food. This is a natural approach, given that in a diet, nutrients and foods eaten together do not act in isolation, but rather show synergistic effects [39]. Therefore, the scope of this systematic review was narrowed down to studies that investigate any link between dietary patterns, components of patients’ diet, and chronic musculoskeletal pain conditions.

### 2.7. Risk of Bias Assessment

The methodological quality of each included study was assessed by two assessors independently (O.E. and S.T.Y.). Risk of bias was assessed using the Cochrane Collaboration’s tool for randomised controlled trials, the standard quality assessment criteria for evaluating primary research for non-randomised controlled trials and uncontrolled clinical trials (QUALSYST), and the Newcastle Ottawa scale (NOS) for observational studies. A consensus meeting was held between the two reviewers to discuss possible differences in methodological quality scores. In case of doubts or disagreements between these two reviewers, a third researcher (A.M.) was consulted to make a final decision.

## 3. Results

### 3.1. Study Selection

**Figure 1:**
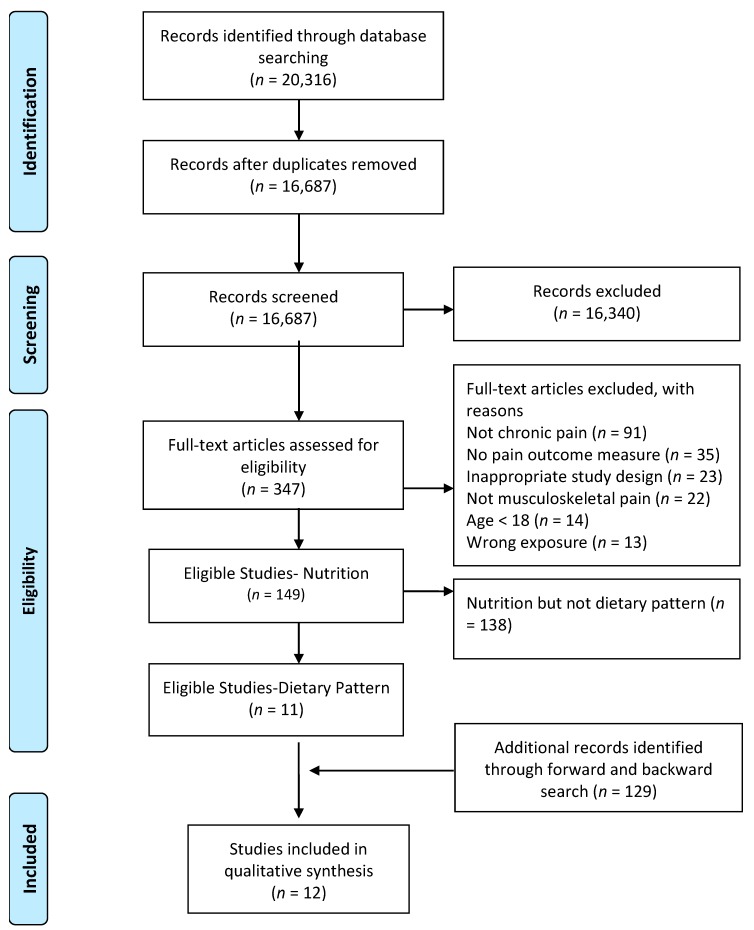
A flowchart giving a detailed overview of the study selection process.

A flowchart giving a detailed overview of the study selection process can be found in Figure 1. From the initial search of the online databases, PubMed, Web of Science, and Embase, 20,316 potentially eligible articles were identified. After removing the duplicates, titles, and abstracts of 16,687 articles were screened and 347 articles were found eligible for full text screening. A total of 149 articles investigated the link between nutrition and chronic musculoskeletal pain conditions, of which 11 focused on overall diet analysis and dietary patterns. Backward and forward tracking resulted in one extra article. Finally, 12 articles were found eligible to be included in the present systematic review.

### 3.2. Study Characteristics

Key data from the included studies are shown in Table 1. The 12 eligible papers included five randomised controlled trials [52,53,54,55,56], one non-randomised controlled trial [57], three uncontrolled clinical trials [58,59,60], one case–control study [49], and two cross-sectional studies [50,51].

In total, four different chronic musculoskeletal pain conditions were studied: osteoarthritis (*n* = 3) [50,52,54], rheumatoid arthritis (*n* = 4) [51,53,55,59], fibromyalgia (*n* = 4) [49,56,57,58], and general musculoskeletal pain (*n* = 1) [60].

Investigated types of dietary patterns in the experimental studies included weight loss diet (*n* = 2) [52,54], peptide diet (*n* = 1) [53], aspartame eliminated diet (*n* = 1) [56], vegetarian diet (*n* = 2) [55,60], vegan diet (*n* = 2) [57,59], and FODMAP (low fermentable oligo-di-mono saccharides and polyols) diet (*n* = 1) [58]. Additionally, in the three observational studies [49,50,51], dietary patterns of patients were obtained, and characteristics of these patterns were explored.

### 3.3. Risk of Bias Assessment

Details of the risk of bias assessment are illustrated in Table 2, Table 3 and Table 4. Using the Cochrane Collaboration’s tool, only one of the five randomised controlled trials was rated as having good quality [52,53,54,55,56], one was rated as fair quality [53], and three were scored as poor quality [52,55,56]. Using the QUALSYST tool for non-randomised controlled trials and uncontrolled clinical trials, two studies scored 0.90 [58,60], whereas two other studies were rated as 0.73 and 0.79 on the total score of 1.00 [57,59]. Using the Newcastle Ottawa risk of bias assessment tool, both cross-sectional studies were rated with five out of seven stars [50,51]. The only case–control study in this review was scored with seven out of nine stars [49].

### 3.4. Results of Individual Studies

This systematic review included nine experimental studies and three observational studies. The results of the individual studies are explained below in detail.

#### Results from Experimental Studies: Effects of Dietary Pattern Change (i.e., Intervention) on Chronic Musculoskeletal Pain

##### (1) Vegetarian Diet

Two studies investigated the effect of a vegetarian diet on chronic musculoskeletal pain [55,60]. A randomised controlled trial including people with rheumatoid arthritis (*n* = 16) applied a lactovegetarian diet for 9 weeks following a 7 to 10 day fasting period instead of the normal, omnivorous diet [55]. During the fasting period, patients were given 800 kcal obtained from 3 litres of fruit and vegetable juices. Participants were not allowed to consume animal or fish protein (including eggs), alcohol, tobacco, coffee, or tea. Intake of salt, sugar, white flour, fresh milk, and cream were also discouraged. 

Although five people with rheumatoid arthritis showed a significant improvement in visual analogue scale (VAS) pain score compared to baseline measurements after the fasting period, there was no statistically significant improvement in pain after the implementation of the lactovegetarian diet compared to the control group [55].

The second study was an uncontrolled clinical trial that focused on the effect of a lacto-ovo vegetarian diet on pain in people with general chronic musculoskeletal pain [60]. This diet consisted of grains, fruits, vegetables, legumes, dairy products and eggs. However, participants were not allowed to eat meat, poultry, seafood, or fish. Processed food and beverages were also discouraged. After an 8 week lacto-ovo vegetarian diet, participants showed significant improvements in pain, as measured by both the numerical pain rating scale (NPRS) and the Short Form 36 (SF-36) pain score compared to baseline measurement [60].

In conclusion, there was some evidence that the implementation of a lacto-ovo vegetarian diet might be effective in decreasing pain in patients with general chronic musculoskeletal pain (level of evidence C and strength of conclusion 3). Conversely, there was some evidence that a lacto-ovo vegetarian diet has no effect on pain in patients with rheumatoid arthritis (level of evidence B and strength of conclusion 3) (please see Table 5 and Table 6 for level of evidence and conclusion).

##### (2) Vegan Diet

Two studies used the vegan diet as dietary pattern intervention [57,59]. First, a non-randomised controlled trial compared a 3 month vegan diet with an omnivorous diet for its effect on pain in people with fibromyalgia [57]. In this study, participants followed one form of vegan diet consisting of uncooked foods, fruits, berries, vegetables, mushrooms, nuts, seeds, legumes, and cereals (living food). The VAS pain score decreased significantly in response to the 3 month vegan diet compared to the omnivorous diet. Moreover, this significant improvement in pain disappeared gradually immediately after shifting back to the omnivorous diet [57].

The second study was an uncontrolled clinical trial including people with rheumatoid arthritis who changed their regular diet to a low-fat vegan diet for 4 weeks [59]. The low-fat vegan diet contained no animal products or added fats and oils of any kind. Compared to baseline measurements, the VAS pain score showed a significant improvement in response to the low-fat vegan diet [59].

In conclusion, there was some evidence that a vegan diet might alleviate chronic musculoskeletal pain (level of evidence B and strength of conclusion 3).

##### (3) Weight Loss Diet

Two studies examined the effect of a weight loss diet on pain [52,54]. The first randomised controlled trial reported a significant decrease in VAS and Western Ontario and McMaster Universities Osteoarthritis Index (WOMAC) pain scores in people with osteoarthritis in response to a hypo-energetic (1200–1400 kcal/day) diet compared to baseline values after 6 months and 1 year [52]. The second randomised controlled study compared a very low energy diet (415 kcal/day) to a low energy diet (810 kcal/day) in osteoarthritis pain [54]. After 8 weeks of intervention, both groups followed a hypo-energetic diet (1200 kcal/day) for 8 more weeks. It was reported that, although there was no significant difference between groups, both groups showed significant improvements in VAS pain score [54].

In conclusion, there was moderate evidence that a hypo-energetic diet might decrease pain severity in patients with chronic osteoarthritis pain (level of evidence A2 and strength of conclusion 2).

##### (4) Peptide Diet

In a randomised controlled trial, the effect of a (commercialised) liquid peptide diet on rheumatoid arthritis pain was investigated [53]. Daily caloric intake of the participants was adjusted to the 30 kcal/kg individually. Participants were not allowed to eat and drink anything else during the 4 week liquid peptide diet except for (soda) water. The average VAS pain score improved significantly after 4 weeks of dietary intervention, but this improvement disappeared at the 3 months follow-up from the end of the 4 weeks of intervention.

In conclusion, there was limited evidence that the liquid peptide diet might decrease pain severity in the short term, but not in the long term, in patients with rheumatoid arthritis (level of evidence B and strength of conclusion 3).

##### (5) Monosodium Glutamate and Aspartame Eliminated Diet

One randomised controlled study investigated the effect of a 3 month monosodium glutamate and aspartame eliminated diet in people with fibromyalgia compared with a normal dietary pattern [56]. Participants in the intervention and control group did not show any significant difference in numerical pain rating score.

In conclusion, there was no evidence that a diet without monosodium glutamate and aspartame has an effect on chronic musculoskeletal pain in patients with fibromyalgia (level of evidence B and strength of conclusion 3).

##### (6) FODMAP Diet

In an uncontrolled clinical trial, people with fibromyalgia followed a low fermentable oligo-di-monosaccharides and polyols (FODMAP) diet for 8 weeks [58]. After the intervention, VAS pain scores significantly decreased.

In conclusion, there was weak evidence that a FODMAP diet might alleviate pain severity in patients with fibromyalgia (level of evidence B and strength of conclusion 3).

Results from observational studies: Dietary pattern characteristics of patients with chronic musculoskeletal pain.

This systematic review included three observational studies: one case–control study, and two cross-sectional studies. The case–control study compared the dietary pattern characteristics and pressure pain thresholds of people with fibromyalgia with healthy, pain-free participants by using a 3 day food diary [49]. Results showed that, compared to the healthy pain free group, the dietary patterns of people with fibromyalgia had a significantly lower caloric intake and intake of carbohydrates, proteins, lipids, vitamin A, vitamin E, vitamin K, folate, selenium, and calcium. There was no significant difference in intake of iron. Regarding the association between pain and diet, a significant positive correlation was found between protein intake and pressure pain thresholds among patients with fibromyalgia (Spearman correlation coefficient = 0.358 and *p* = 0.018).

The first cross-sectional study analysed the dietary patterns of people with rheumatoid arthritis by using a 3 day food diary and comparing it with dietary reference intake values [51]. Additionally, associations between VAS score of pain and nutrient intake were considered. Results of the dietary pattern analysis indicate that the intake of energy and micronutrients including calcium, folic acid, zinc, magnesium, and vitamin B6 were considerably lower in people with rheumatoid arthritis compared to the dietary reference values. On the other hand, in most of the patients, intake of protein, copper, and vitamin E met or exceeded the recommended dietary reference value [61]. Importantly, pain severity did not show any significant association with nutrient intake.

The second cross-sectional study used the VAS pain score and a 2 day food diary in people with osteoarthritis [50]. Among obese and overweight people with osteoarthritis who had chronic pain, pain severity was positively associated with calorie and fat intake. Patients having severe pain (VAS pain score > 7) reported significantly more intake of sugar and fat compared to those having low pain severity (VAS pain score between 0 to 2).

To conclude, observational studies mainly evaluated the nutritional status of the patients with chronic musculoskeletal pain by comparing their dietary patterns with the dietary patterns of healthy, pain-free people or by comparing their data with nutritional reference values. To summarize, it is suggested that the intake of energy, calcium, folic acid, zinc, magnesium, and B6 is lower than the dietary reference values in patients with chronic rheumatoid arthritis pain [61] (level of evidence C and strength of conclusion 3) and intake of calorie, proteins, carbohydrates, lipids, vitamin A, vitamin K, folate, selenium, and calcium is lower in patients with chronic fibromyalgia pain compared to the nutritional intake of healthy, pain-free people (level of evidence B and strength of conclusion 3). Pain threshold was also found to be positively associated with protein intake in patients with chronic fibromyalgia, and pain severity was found to be positively associated with the intake of sugar and fat in patients with chronic osteoarthritis pain (level of evidence B and strength of conclusion 3).

## 4. Discussion

The aim of this systematic review was to investigate the association between dietary patterns and chronic musculoskeletal pain conditions. The search strategy led to the inclusion of 12 papers, including nine experimental and three observational studies. Four main approaches were used to investigate the association between dietary patterns and chronic musculoskeletal pain: (1) the effects of a specific dietary pattern intervention on pain in patients with chronic musculoskeletal pain; (2) comparing the dietary patterns and nutritional intakes of patients having chronic musculoskeletal pain with those of healthy, pain free-controls; (3) comparing dietary patterns and nutrient intakes of patients with chronic musculoskeletal pain with standard dietary reference intake values; and (4) analysing the dietary patterns of patients with chronic musculoskeletal pain cross-sectionally and investigating the association between pain severity and food/nutrient intake.

Regarding the experimental studies, seven out of nine experimental studies reported improvement in chronic pain in response to a specific diet [52,53,54,55,56,57,58,59]. These positive, pain-reducing diets included a vegan diet for fibromyalgia [57] and rheumatoid arthritis [59], weight loss diet for osteoarthritis [52,54], a vegetarian diet for general musculoskeletal pain [60], a FODMAP diet for fibromyalgia [58], and a peptide diet for rheumatoid arthritis [53]. On the other hand, two studies out of nine did not find any effect of a dietary pattern intervention on pain (i.e., the monosodium glutamate and aspartame eliminated diet for fibromyalgia [56] and the vegetarian diet for rheumatoid arthritis [55]).

Among the studies that found positive effects of dietary changes on pain intensity, improvement in pain may be explained because of an increase in healthy eating compared to the regular dietary pattern. Four out of nine studies used a vegetarian or vegan diet. According to the Healthy Eating Index-2010 and the Mediterranean diet score, which allow objectivation of overall diet quality [62], these plant-based diets are considered more healthful compared to an omnivorous diet. Additionally, according to the Dietary Inflammatory Index (DII) which was developed to measure the inflammatory potential of specific foods and dietary constituents [63], plant-based dietary patterns are classified as anti-inflammatory dietary patterns [64]. Therefore, more specifically, pain-relieving effects of vegetarian and vegan diets might result from their anti-inflammatory characteristics. Chronic inflammation is often suggested as one of the mechanisms underlying chronic musculoskeletal pain disorders such as chronic low back pain [65], rheumatoid arthritis, fibromyalgia [66], myofascial pain syndrome [67], osteoarthritis [68], and work-related overuse syndrome [69]. During the inflammatory response, tissue releases several inflammatory biomarkers that activate nociceptors [70,71]. If the presence of these inflammatory biomarkers persists beyond the normal healing time, this leads to pain chronification due to prolonged peripheral sensitization, changes in peripheral and central neuronal structure, and central sensitization [71,72]. To give an example, myofascial trigger points defined as hyperirritable nodules within taut bands of skeletal muscle have been associated with numerous musculoskeletal pain conditions such as myofascial pain syndrome [67], lower back pain [73], neck pain [74], and fibromyalgia [75]. The active trigger points can cause an inflammatory response and lead to release of inflammatory biomarkers at the surrounding muscles locally and even at the remote uninvolved tissues [76]. Presence of this inflammatory response caused by active painful trigger points can constitute a structural pathologic source of persistent nociception leading to peripheral and central sensitization [77,78].

Vegetarian and vegan diets often consist of less protein, sugar, carbohydrate, and caloric intake compared to an omnivorous diet, and are therefore more in line with dietary recommendations for healthy eating [62]. Similar to Mediterranean and Palaeolithic diets, vegetarian and vegan diets are fruit and vegetable-based dietary patterns and are inversely associated with the presence of inflammatory biomarkers [44,62,79]. Conversely, a higher intake of protein, fat, sugar, and calories shows a positive correlation with pain intensity and are characteristic features of the Western diet, which is associated with chronic low-level inflammation [44], and thus with chronic pain and pain intensity [71].

It is also reported that compared to an omnivorous or Western style diet, plant-based diets such as the vegetarian, vegan, and Mediterranean diets play important roles in protecting the stability and diversity of the gut microbiome [80]. The reason behind the interaction between plant-based diets and healthy gut microbiome is the high proportion of some specific nutrients such as high amounts of dietary fibre, mono- and polyunsaturated fatty acids, plant-derived proteins, and polyphenols [80]. There is a bi-directional relationship between the central nervous system and gut microbiota, which is called the gut–central nervous system axis [81]. It is concluded that gut microbiome can cause systemic inflammation and inflammation in the central nervous system, which can contribute to pain chronification and amplification [81,82]. Therefore, the application of a more plant-based diets might alleviate chronic musculoskeletal pain by their positive effects on gut microbiome and eventually on systemic and central inflammation.

Available evidence shows that among obese or overweight osteoarthritis patients suffering from chronic pain, higher pain severity is associated with higher calorie and fat intake, and patients with severe pain reported greater intake of sugar and fat [50]. This might also explain the pain improvement in the two randomised controlled studies that explored a calorie-restricted diet [52,54]. However, in both studies, patients significantly lost weight after the calorie-restricted diet compared to baseline measurements. Therefore, the decrease in BMI, more specifically decrease in fat mass, might also be a reason for the pain decrease, as obesity itself can also negatively impact pain, given the low-level chronic inflammation it may cause, as the adipose tissue can release proinflammatory cytokines [83]. Additionally, the decrease in fat mass and eventually in chronic inflammation might also lead to a long-term pain-relieving effect on chronic musculoskeletal pain. This might constitute an effective treatment modality for patients with chronic musculoskeletal pain who cannot reach adequate pain relive effects with drugs and develop side effects [84,85].

Interestingly, in this review, we could not identify eligible studies that investigated the Mediterranean diet. The Mediterranean diet comprises consumption of olive oil as a main source of fat, as well as vegetables, fruits, legumes, cereals, and fish [86]. Compared to the Western diet, the Mediterranean diet consists of more fibre [87], antioxidants [88,89], and α-linolenic acid [90], and less linoleic acid [91,92], which probably gives it anti-inflammatory characteristics [92]. The pain-relieving effect of this diet may be due to its anti-inflammatory effects. The Mediterranean diet shows an inverse relation with the systemic inflammation level [93]—lower scores on the Mediterranean diet indicate a pro-inflammatory dietary intake [43], and people who exhibit a higher adherence to the Mediterranean Diet show lower scores on the dietary inflammatory index, which means that characteristics of their diet are more anti-inflammatory [64,94]. There are some studies suggesting the Mediterranean diet as reducing pain. For instance, in their systematic review, Forsyth et al. found evidence for pain reduction among people with rheumatoid arthritis following the Mediterranean diet [95]. However, studies that examined the Mediterranean diet as an intervention included both patients without pain syndrome and patients with acute pain, and were therefore excluded from the present review [96].

Results from the included observational studies, in which dietary patterns of the patients were analysed, mainly indicated that, among patients with chronic musculoskeletal pain, intake of nutrients can be below the dietary reference values [51] and less than the nutritional intake of healthy, pain-free people [49]. The intake of nutrients such as fat, sugar, and protein are associated with the severity of chronic pain [49,50]. However, these findings differ among the chronic musculoskeletal pain conditions. For instance, protein intake was associated with pain thresholds in fibromyalgia [49], whereas fat and sugar intake were associated with pain severity in patients with chronic osteoarthritis pain [50].

Among patients with fibromyalgia, a significant positive association between protein intake and pain threshold was reported [49]. Batista et al. reported a lower intake of carbohydrates, proteins, lipids, vitamin A-E-K, folate, selenium, and zinc in patients with fibromyalgia compared to healthy women without fibromyalgia [49]. As is reported in this study, inadequate protein intake might contribute to the fibromyalgia pain by tryptophan deficiency, which might contribute to pain among patients with fibromyalgia via the tryptophan-serotonin metabolism [38]. A positive association between protein intake and pain threshold is another possible sign of association between protein malnutrition and pain in fibromyalgia patients [49]. In their meta-analysis, Joustro et al. found that patients with fibromyalgia had significantly lower serum levels of vitamin E compared to healthy participants [97]. Therefore, as Batista et al. reported, less vitamin E intake [49] and inadequate intake of certain vitamins and minerals might play a role in the pathophysiology of fibromyalgia pain.

On the other hand, in patients with rheumatoid arthritis, pain severity did not show any significant association with nutrient intake. It is suggested that patients diagnosed with rheumatoid arthritis who have chronic pain have significantly less intake of calcium, folic acid, zinc, magnesium, and vitamin B6 compared to reference values [51,61]. However, in patients diagnosed with rheumatoid arthritis, it is possible that patients do not have chronic pain. Studies that included patients without chronic pain were excluded from this review. Thus, in this group of patients, the appearance of pain and pain severity might become less relevant with the dietary intakes because of the nature and underlying mechanism of the disease.

### 4.1. Strengths and Limitations

There are several limitations to consider when reading this systematic review. A first limitation is the generally low methodological quality of the included studies. Results discussed in this review mainly relied on observational research, uncontrolled clinical trials, and non-randomised clinical trials. Additionally, all included experimental studies were single-blinded, which means patients were aware of the intervention. Selection of the participants was done according to the willingness of the patients to participate. This increases the odds of selection bias, as it is possible that psychological factors such as treatment expectations may play a role in explaining the observed pain relief in response to dietary pattern changes. For instance, it was reported that the vegetarian diet is more effective on pain reduction among patients with rheumatoid arthritis if the individuals expect that dietary change and complementary therapies will improve their pain [98]

A second limitation is the heterogeneity of the participants’ characteristics. In total, five different chronic musculoskeletal pain conditions were studied in the included studies. Therefore, underlying pain-generating mechanisms might differ among various chronic musculoskeletal conditions. For instance, although it was reported that fat and caloric intake is associated with pain severity in patients with osteoarthritis [50], fat and caloric intake were not associated with pain severity in patients with rheumatoid arthritis [51]. This might be due to different underlying pain generator mechanisms [16]. On the other hand, the primary aim of this review was to show all possible interactions between dietary pattern, components of patients’ diet, and chronic musculoskeletal pain. Therefore, at this early stage of the new research line, inclusion of all the chronic musculoskeletal pain conditions provides a more holistic view of the available literature on the link between nutritional factors and chronic musculoskeletal pain.

A third limitation of this study is that, except for one study [49], assessments of pain as an outcome was performed using self-reported unidimensional pain measurement tools such as VAS and NPRS. Only one study used a pressure pain threshold algometer to measure the relation between dietary intake and pain threshold [49]. Although these unidimensional scales are easily applicable and are commonly used tools for measuring acute pain, multidimensional pain rating scales are suggested for chronic pain [99]. VAS and NPRS depend on the patients’ experiences of pain, and are therefore highly subjective [100]. Patients cannot be blinded to the assessment of the effects of the dietary interventions on pain. Thus, it is possible that the overestimation of the patients might unconsciously contribute to the results of the study [101].

Lastly, this study did not include a meta-analysis. Initially we intended to perform a meta-analysis. However, due to the heterogeneity of the included studies, only qualitative analysis of the studies was possible. Variety in study design, dietary intervention, and chronic musculoskeletal pain conditions were major sources of study heterogeneity, precluding a meta-analytic approach to the available data. Only five randomised controlled trials were included. These five studies consisted of three different chronic musculoskeletal pain conditions and applied four different diet types as intervention [52,53,54,55,56].

However, several strengths of this review are worth mentioning. First, this systematic review is the first of its kind to investigate the relationship between dietary patterns, components of patients’ diet, and pain in patients with chronic musculoskeletal pain. Second, each step in this review, including an online database search, study selection, risk of bias assessment, and data extraction, was performed by two independent reviewers. This enabled the prevention of detection and performance bias. Third, the systematic review was registered a priori in the PROSPERO database, and adhered to international standards for the conducting and reporting of systematic reviews [47]. Finally, the systematic review was supervised by an interdisciplinary team of researchers combining the necessary expertise from the various fields needed to study the interaction between nutrition/diet and chronic pain.

### 4.2. Recommendations for Further Research

Most of the included studies were methodologically low in quality. Although there were five randomised controlled trials included in this review, only one of them was rated as having a low risk of bias. Therefore, more rigorous and high-quality clinical trials are urgently needed.

The heterogeneity of the studied chronic musculoskeletal pain conditions and variety in dietary interventions made it impossible to have a clear view of the mechanisms that play a role in the association between nutrition and pain. It is unfeasible to conduct a systematic review on a specific musculoskeletal pain condition due to an insufficient number of researches in a specific musculoskeletal pain group. Thus, more studies on each musculoskeletal pain condition are needed.

Studies that focus on the causal relation between systemic inflammation, chronic musculoskeletal pain conditions, and dietary patterns are needed to explain the underlying mechanisms.

## 5. Conclusions

On the basis of the available literature, there is some evidence that plant-based dietary patterns such as vegetarian and vegan diets might have pain-relieving effects on chronic musculoskeletal pain. This effect might arise from the anti-inflammatory characteristics of the plant-based dietary patterns, but studies exploring the mechanisms behind the pain-relieving effects of dietary interventions for patients with chronic musculoskeletal pain are needed. There is also inconclusive evidence that patients may show inadequate intake of calcium, folate, zinc, magnesium, and vitamin B6 in chronic rheumatoid arthritis pain and a lower intake of carbohydrates, proteins, lipids, vitamin A-E-K, folate, selenium, and zinc in chronic fibromyalgia pain compared to the reference values and data of healthy people, respectively. Moreover, pain severity is positively associated with fat and sugar intake in chronic osteoarthritis pain, and pain threshold is positively associated with protein intake. However, the mechanisms behind this interaction are still uncertain, and more high-quality studies that investigate the underlying mechanisms of the interaction between chronic musculoskeletal pain and nutrition are needed.

## Figures and Tables

**Table 1:** Data extraction table.

Author (Year) (Reference) and Condition	Design and Duration	Participant Number (Female), Age, Body Mass Index	Intervention (or Case) Group	Control Group	Outcome Measures	Findings
**Observational Studies**						
Batista et al. (2016) [49] and FB	CC	**FB:** 43 (43 F) 49 ± 7.92 26.96 ± 4.64 **C:** 44 (44 F) 46.8 ± 10.36 25.72 ± 3.76	Three-day dietary record was used to find out intake of total calories, carbohydrates, lipids, vitamins (A, C, B12, D, and K), and minerals (folate, selenium, zinc, iron, calcium, and magnesium). Additionally, in order to investigate the association between nutrient intake and pain severity, pain pressure threshold was used, and measurement area was right trapezius muscle.	Healthy people	Three-day food diary PPTs	Healthy control group showed significantly higher caloric intake and intake of carbohydrates, proteins, lipids, vitamin A, vitamin E, vitamin K, folate, selenium, and calcium. There was no significant difference in intake of iron. Additionally, there was only a significant positive correlation between protein intake and pain.
Choi et al. (2014) [50] and OA	CS	**OA:** 54 (49 F) 62.7 ± 9.3 32.6 ± 4.8	Two-day dietary record used in order to measure the food intake of patients. Additionally, VAS pain scale used in order to assess the pain intensity and its relationship with food and nutrient intake.	No control group	Food diary VAS	Among obese or overweight OA patients who have chronic pain, pain severity is positively correlated with calorie and fat intake. Patients who have severe pain reported more intake of sugar and fat.
Hejazi et al. (2011) [51] and RA	CS	**I:** 90 (90 F) 47.47 ± 13.68 23.9 ± 1.59	Patients’ dietary and nutrient intakes were analysed and compared with the standard dietary reference intake values.	No control group	Three-day food diary VAS	Intake of energy and micronutrients including calcium, folic acid, zinc, magnesium, and vitamin B6 were considerably lower compared with the dietary reference values. On the other hand, intake of protein, copper, and vitamin E met or exceeded the recommended dietary reference value in most of the patients. Pain severity did not show any significant correlation with any nutrient intakes.
**Interventional Studies**						
Bellare et al. (2014) [52] and OA	RCT and 1 year	**I:** 61 (50 F) 59.98 ± 8.81 27.36 ± 3.71 **C:** 56 (40 F) 60.70 ± 8.31 27.68 ± 3.03	**Weight loss diet alone and with supplement:** Intervention group, diet, and supplement group were supplemented with glucosamine (1500 mg) and chondroitin (1200 mg) sulphate per day, in addition to the same diet therapy as diet-only group (weight lose diet). Control group and diet-only group received weight loss diet which is a balanced energy-controlled diet supplying 1200–1400 kcal per day (carbohydrate 50%–55%, protein 15%–25%, fat < 30%).	Weight loss diet alone	VAS (0 to 10) WOMAC pain	A total of 16 patients from diet-only group and 12 patients from diet and supplementation group withdrew. Both diet-only and diet with supplement group showed significant decrease in pain after 6 months and 1 year (*p* ≤ 0.05). However, pain better improved according to the VAS and WOMAC scores in the supplement group after 1 year. Additionally, in the first 6 months, improvements in pain were faster than the second 6 months.
Holst-Jensen (1998) [53] and RA	RCT and 6 months	**I:** 15 (10 F) Mean age = 56 (range 34–70) BMI = NR **C:** 15 (14 F) Mean age = 46 (range 29–72) BMI = NR	**eptide diet:** Patients were explained the artificial elemental diet and were given a commercialised liquid diet (TU). The liquid diet contained soy protein, methionine, tryptophan, vitamins, and trace elements. The diet lasted 4 weeks and then patients were followed for 6 months in total from the baseline until the end. During the peptide diet, patients were not allowed to eat and drink any other foods or beverages except for water and plain soda water.	Normal food intake	VAS (0 to 10)	A total of 2 and 1 patients withdrew from the intervention and control group, respectively. Pain now, average pain, and worst pain during the last week were measured. Only average pain during the last week significantly improved after the 4 weeks of intervention from 5(1.4/7.0) to 4(1.4/6.6) (*p* = 0.02). However, this effect disappeared at the 3 month follow up, and there was no between-group differences.
Riecke et al. (2010) [54] and OA	RCT and 16 weeks	**I:** 96 (78 F) 61.8 ± 6.4 37.5 ± 5.4 **C:** 96 (77 F) 63.3 ± 6.3 37.1 ± 4.1	**Very low energy diet:** Patients (BMI > 30 kg/m^2^) were expected to follow a very low energy diet (415 kcal/day) in the intervention group and were expected to follow a low energy diet (810 kcal/day) in the control group for 8 weeks. After that, both groups followed a hypo-energetic diet (1200 kcal/day) for a second 8 weeks.	Low-energy diet	VAS (0 to 100)	A total of 10 patients from the very low energy diet group and 7 patients from the low energy diet group withdrew. Pain significantly decreased in both groups after 16 weeks, with a pooled average for pain 9.72 (95% CI: 7.72–11.72; *p* < 0.001). However, there were no significant difference between the groups.
Sköldstam, Larsson, and Lindström (1979) [55] and RA	RCT and 10 weeks	**I:** 16 (10 F) Mean Age = 52 (range = 35–66) BMI = NR **C:** 10 (9 F) Mean age = 54 (range = 43–65) BMI = NR	**Fasting and lactovegetarian diet:** After fasting for 7–10 days, participants followed a lactovegetarian dietary pattern for the following 9 weeks with a 1 week resting period between fasting and vegetarian diet. During fasting, daily energy supply was 800 kJ. During the lactovegetarian period, no animal or fish protein, egg, alcohol, tobacco, coffee, or tea allowed.	Normal diet	VAS (0 to 10)	One patient during the fasting and one patient during the vegetarian diet withdrew from the study. After fasting, only five patients showed significant improvement in pain. However, there was no significant difference in pain after the lactovegetarian dietary pattern.
Vellisca and Latorre (2014) [56] and FB	RCT and 3 months	**I:** 36 (36 F) 42.33 ± 8.43 NR **C:** 36 (36 F) 39.64 ± 8.16 NR	**Monosodium glutamate and aspartame eliminated diet:** Patients were educated to detect and eliminate the monosodium glutamate and aspartame from their diet. They were expected to follow this diet for 3 months.	Normal diet	NPRS (0 to 7)	Monosodium glutamate and aspartame eliminated diet did not show a significant effect compared to normal dietary pattern (*p* = 0.178).
Kaartinen et al. (2000) [57] and FB	NCT and 3 months	**I:** 18 (18 F) mean age = 51 mean BMI = 28 **C:** 15 (15 F) mean age = 52 mean BMI = 28	**Vegan diet:** Patients were educated to prepare their diet according to the rules of the dietary pattern for 3 months. After 3 months, they continued their normal omnivorous diet, the diet that they wanted to follow.	Omnivorous Diet	VAS	The results revealed significant improvements in visual analogue scale of pain after 3 months of vegan diet (*p* = 0.005). However, this significant improvement disappeared after shifting to omnivorous diet gradually.
Marum et al. (2017) [58] and FB	UCT and 8 weeks	**I:** 38 (38 F) mean age = 51 27.4 ± 4.6	**A low fermentable oligo-di-monosaccharides and polyols (FODMAP) diet:** Subjects were educated according to the FODMAP diet and were expected to follow this diet as an intervention for 8 weeks.	No control group	VAS (0 to 10)	Seven participants withdrew from the study. Pain measurement showed a statistically significant decrease after the fourth week. This significance did not exist between the fourth and eighth week. However, compared to baseline, pain significantly decreased after 8 weeks (week 0 = 6.6, week 4 = 4.9, week 8 = 5.4) (*p* < 0.01).
McDougall et al. (2002) [59] and RA	UCT and 4 weeks	**I:** 24 (23) 56 ± 11 NR	**Very low fat vegan diet:** Four meetings held in order to teach a low-fat vegan diet to the participants. They were expected to follow a low-fat vegan diet for 4 weeks. The diet contained no animal products or added fats and oils of any kind.	No control group	VAS (0 TO 100)	No one withdrew from the study. Pain significantly decreased compared to baseline from 49 ± 20 to 34 ± 20 (*p* < 0.04).
Towery et al. (2018) [60] and MSK	UCT and 8 weeks	**I:** 14 (12 F) 48.07 ± 16.92 36.13 ± 10.45	**Lacto-ovo vegetarian diet:** Patients have been given an education on plant-based diet and were expected to follow this dietary pattern for 8 weeks. Meat, poultry, seafood, and fish were not allowed.	No control group	NPRS (0 TO 10) SF-36 pain	No drop outs. Pain rating significantly decreased with an average of 3.14 point in NPRS (*p* = 0.0001). Pain rate in SF-36 significantly decreased an average of 25.53 points (*p* = 0.0001).

MSK, musculoskeletal pain; FB, fibromyalgia; OA, asteoarthritis; RA, rheumatoid arthritis; RCT, randomised controlled trial; NCT, non-randomised controlled trial; UCT, uncontrolled clinical trial; CC, case control study; CS, cross sectional study; C, control group; I, intervention group; PPTs, pressure pain threshold; VAS, visual analogue scale; WOMAC, The Western Ontario and McMaster Universities Index; NPRS, numerical pain rating scale; BMI, body mass index; Kcal, kilo calorie; Mg, milligram; NR, not reported; F, female.

**Table 2:** Risk of bias assessment of randomised controlled trials (*n* = 5).

Author (Year)	Selection Bias		Performance Bias	Detection Bias	Attrition Bias	Reporting Bias	Other Bias	Total
	Random Sequence Generation	Allocation Concealment	Blinding of Participants and Personnel	Blinding of Outcome Assessment	Incomplete Outcome Data	Selective Reporting	Anything Else, Ideally Prespecified	Good Fair Poor
Bellare et al. (2014) [52]	Unclear	Unclear	High	High	Unclear	Low		Poor
Riecke et al. (2010) [54]	Low	Low	Low	Low	Low	Low		Good
Holst-Jensen (1998) [53]	Low	Low	Unclear	Unclear	Low	Low		Fair
Sköldstam, Larsson, and Lindström (1979) [55]	Unclear	Unclear	Unclear	Unclear	High	Low		Poor
Vellisca and Latorre (2014) [56]	Unclear	Unclear	Unclear	Unclear	Low	Low		Poor
**Selection Bias**								
- Criterion 1: Selection bias (biased allocation to interventions) due to inadequate generation of a randomised sequence.								
- Criteria 2: Selection bias (biased allocation to interventions) due to inadequate concealment of allocations prior to assignment.								
**Performance Bias**								
- Criterion 1: Performance bias due to knowledge of the allocated interventions by participants and personnel during the study.								
**Detection Bias**								
- Criterion 1: Detection bias due to knowledge of the allocated interventions by outcome assessors.								
**Attrition Bias**								
- Criterion 1: Attrition bias due to amount, nature, or handling of incomplete outcome data.								
**Reporting Bias**								
- Criterion 1: Reporting bias due to selective outcome reporting.								
**Other Bias**								
- Criterion 1: Bias due to problems not covered elsewhere in the table.								

**Table 3:** Risk of bias assessment of non-randomised controlled and uncontrolled clinical trials.

ASSESSMENT CRITERIA Non-Randomised Controlled Trials Uncontrolled Clinical Trials	Kaartinen et al. (2000) [57]	Marum et al. (2017) [58]	Towery et al. (2018) [60]	McDougall et al. (2002) [59]
1. Question/objective sufficiently described?	YES	YES	YES	YES
2. Study design evident and appropriate?	YES	YES	PARTIAL	PARTIAL
3. Method of subject/comparison group selection or source of information/input variables described and appropriate?	PARTIAL	YES	YES	YES
4. Subject (and comparison group, if applicable) characteristics sufficiently described?	YES	YES	YES	YES
5. If interventional and random allocation was possible, was it described?	NO	N/A	N/A	N/A
6. If interventional and blinding of investigators was possible, was it reported?	NO	N/A	N/A	PARTIAL
7. If interventional and blinding of subjects was possible, was it reported?	N/A	N/A	N/A	N/A
8. Outcome and (if applicable) exposure measure(s) well defined and robust to measurement/misclassification bias? Means of assessment reported?	YES	YES	YES	YES
9. Sample size appropriate?	PARTIAL	PARTIAL	PARTIAL	PARTIAL
10. Analytic methods described/justified and appropriate?	YES	YES	YES	YES
11. Some estimate of variance is reported for the main results?	YES	YES	YES	PARTIAL
12. Controlled for confounding?	PARTIAL	YES	YES	PARTIAL
13. Results reported in sufficient detail?	YES	YES	YES	YES
14. Conclusions supported by the results?	YES	YES	YES	YES
**TOTAL**	**0.73**	**0.90**	**0.90**	**0.79**

**Table 4:** Risk of bias assessment of case–control and cross-sectional studies.

Author (Year) and Study Design	Selection				Comparability		EXPOSURE for Case Control Studies/or OUTCOME for Cross Sectional Studies			Total Stars
	S1	S2	S3	S4	C1	C2	E1/O1	E2/O2	E3	
Choi et al. (2014) [50] and cross-sectional	*	*	-	N/A	*	*	-	*	N/A	5/7
Hejazi et al. (2011) [51] and cross-sectional	*	*	*	N/A	*	*	-	*	N/A	5/7
Batista et al. (2016) [49] and case–control	*	-	*	*	*	*	*	*	-	7/9
**Selection**										
- S1 for case–control studies; is the case definition adequate?										
- S1 for cross-sectional studies; representativeness of the sample.										
- S2 for case–control studies; representativeness of the cases.										
- S2 for cross-sectional studies; non-respondents.										
- S3 for case–control studies; selection of the controls.										
- S3 for cross-sectional studies; ascertainment of the exposure.										
- S4 for case–control studies; definition of the control.										
**Comparability**										
- C1 for case–control studies; study controls for most important factor.										
- C1 for cross-sectional studies; study controls for most important factor.										
- C2 for case–control studies; study controls for any additional factors.										
- C2 for cross-sectional studies; study controls for any additional factors.										
**Exposure**										
- E1; ascertainment of exposure.										
- E2; same method of ascertainment of cases and controls.										
- E3; non-response rate.										
**Outcome**										
- O1; assessment of outcome.										
- O2; statistical analysis.										

**Table 5:** Level of evidence.

Level of Evidence	Intervention
**A1**	Systematic review of at least two studies conducted independently from each other of evidence level A2.
**A2**	Randomised double-blinded comparative clinical research of good quality and efficient size
**B**	Comparative research, but not with all characteristics mentioned for A2. This also includes patient– control research and cohort research.
**C**	Non-comparative research.
D	Opinion of experts.

**Table 6:** Level of conclusion.

Level of Conclusion	Conclusion Based on
**1**	Research of evidence level A1 or at least two independently conducted studies of evidence level A2.
**2**	One research of evidence level A2 or at least two independently conducted studies of evidence level B.
**3**	One research of evidence level B or C.
**4**	Opinion of experts or inconclusive or inconsistent results between various studies.

## Supplementary Materials

The following are available online at https://www.mdpi.com/2077-0383/9/3/702/s1.
