# Supplementary material for: Do Nutritional Factors Interact with Chronic Musculoskeletal Pain? A Systematic Review
Source: J Clin Med. 2020 Mar 5;9(3):702. doi: 10.3390/jcm9030702 (PMC7141322; doi:10.3390/jcm9030702)
Supplement: Supplementary file 1 [file jcm-09-00702-s001.pdf]

SUPPLEMENTARY DOCUMENTS

Supplementary Table 1: Used Search Terms in PubMed

	Keywords
Group-1 (Patient)	“Musculoskeletal Pain” [Mesh] OR “Chronic Pain” [Mesh] OR “Persistent Pain” OR “Joint Pain” OR “Muscle Pain” [Mesh] OR “Bone pain” OR “tendon pain” OR “Ligament pain” OR “Bursa pain” OR “Limb Pain” OR “Hand Pain” OR “Wrist Pain” OR “Elbow Pain” OR “forearm pain” OR “Shoulder Pain” [Mesh] OR “Arm Pain” OR “heel pain” OR “Ankle Pain” OR “Knee Pain” OR “Hip Pain” OR “Pelvic pain” OR “Leg Pain” OR “Spinal Pain” OR “Back Pain” [Mesh] OR “Neck Pain” [Mesh] OR “Whiplash injury” OR “jaw pain” OR “Temporomandibular Disorder*” OR “Widespread pain” OR “Fibromyalgia” [Mesh] OR “Myalgia” [Mesh] OR “Myofascial Pain” OR “Arthralgia” [Mesh] OR “Osteoarthritis” [Mesh] OR “arthritis” [Mesh] OR “Epicondylitis” OR “Tendinitis” OR “Tendinopathy” OR “tenosynovitis” [Mesh] OR “Bursitis” [Mesh] OR “Cervicogenic headache” OR “tension type headache” OR “Migraine headache” OR “Osteoporosis pain”
Group-2 (Exposure)	“Nutrition*” OR “Diet*” [Mesh] OR “Eating Behavio$r” OR “Macronutrient*” OR “Micronutrient*” OR “Fat intake” OR “Fat consum*” OR “fat absorption” OR “fat absorb” OR “fat supplement*” OR “Lipid intake” OR “lipid consum*” OR “Lipid absor*” OR “lipid supplement*” OR “Fatty Acid* intake” OR “Fatty Acid consum*” OR “Fatty Acid absor*” OR “fatty acid supplement*” OR “Omega 3 intake” OR “omega 3 consum*” OR “omega 3 absor*” OR “omega 3 supplement*” OR “Omega 6 intake” OR “omega 6 consum*” OR “omega 6 absor*” OR “omega 6 supplement*” OR “Protein intake” OR “Protein consum*” OR “protein absor*” OR “protein supplement” OR “protein drink” OR “Amino acid intake” OR “amino acid consum*” OR “Amino acid absor*” OR “amino acid supplement*” OR “Carbohydrate intake” OR “carbohydrate consum*” OR “carbohydrate absor*” OR “carbohydrate supplement*” OR “Sugar intake” OR “sugar consum*” OR “sugar absor*” OR “Sucrose intake” OR “sucrose consum*” OR

	“sucrose absor*” OR “Glucose intake” OR “glucose consum*” OR “glucose absor*” OR “Fructose intake” OR “fructose consum*” OR “Fructose absor*” OR “Fibre intake” OR “fibre consum*” OR “fibre absor*” OR “fibre supplement*” OR “Fiber intake” OR “Fiber consum*” OR “fiber absor*” OR “Fiber Supplemet” OR “Starch intake” OR “ starch consum*” OR “starch absor*” OR “Food” [Mesh] OR “Vegetable*” [Mesh] OR “Fruit*” [Mesh] OR “Vegan” [Mesh] OR “Vegetarian” OR “Omnivorous” OR “Carnivorous” OR “Meat” [Mesh] OR “Fish” [Mesh] OR “Legume*” OR “Spice” OR “Spices” OR “Nut” [Mesh] OR “Nuts” OR “Seed*” [Mesh] OR “Whole Grain*” [Mesh] OR “Dairy” [Mesh] OR “Milk” [Mesh] OR “Cheese” [Mesh] OR “Yogurt” [Mesh] OR “Egg” OR “Eggs” OR “Olive” OR “Calorie” OR “Calori* intake” OR “Energy intake” [Mesh] OR “Sweetened beverage” OR “Alcohol” OR “Wine” OR “Caffeine” [Mesh] OR “Coffee” [Mesh] OR “Tea” [Mesh] OR “Water” [Mesh] OR “Soft Drink” OR “Soda” OR “Vitamin intake” OR “vitamin consum*” OR “vitamin absor*” OR “vitamin supplement*” OR “Vitamin drink” OR “Mineral intake” OR “mineral absor*” OR “mineral supplement” OR “mineral drink” OR “Antioxidant*”
Terms “NOT” included	“Acute pain” [Mesh] OR “subacute pain” OR "baby" OR “New Born” OR "Infant" [Mesh] OR “Premature” OR “Postmature“ OR “Infants” OR "P?ediatric" OR "Children" OR "child" [Mesh] OR “Adolescent” OR “Adolescents” OR "preschool" OR “Primary School” OR "Animal study" OR “Animal research” OR “animal experiment*” OR "rat" OR "rats" OR "mice" OR "mouse" OR “Hamster” OR “Hamsters” OR “Rabbit” OR “Rabbits”

Supplementary Table 2: Used Search Terms in Web of Science and Embase

	Keywords
Group-1 (Patient)	“Musculoskeletal Pain” OR “Chronic Pain” OR “Persistent Pain” OR “Joint Pain” OR “Muscle Pain” OR “Bone pain” OR “tendon pain” OR “Ligament pain” OR “Bursa pain” OR “Limb Pain” OR “Hand Pain” OR “Wrist Pain” OR “Elbow Pain” OR “forearm pain” OR “Shoulder Pain” OR “Arm Pain” OR “heel pain” OR “Ankle Pain” OR “Knee Pain” OR “Hip Pain” OR “Pelvic pain” OR “Leg Pain” OR “Spinal Pain” OR “Back Pain” OR “Neck Pain” OR “Whiplash injury” OR “jaw pain” OR “Temporomandibular Disorder*” OR “Widespread pain” OR “Fibromyalgia” OR “Myalgia” OR “Myofascial Pain” OR “Arthralgia” OR “Osteoarthritis” OR “arthritis” OR “Epicondylitis” OR “Tendinitis” OR “Tendinopathy” OR “tenosynovitis” OR “Bursitis” OR “Cervicogenic headache” OR “tension type headache” OR “Migraine headache” OR “Osteoporosis pain”
Group-2 (Exposure)	“Nutrition*” OR “Diet*” OR “Eating Behavio$r” OR “Macronutrient*” OR “Micronutrient*” OR “Fat intake” OR “Fat consum*” OR “fat absorption” OR “fat absorb” OR “fat supplement*” OR “Lipid intake” OR “lipid consum*” OR “Lipid absor*” OR “lipid supplement*” OR “Fatty Acid* intake” OR “Fatty Acid consum*” OR “Fatty Acid absor*” OR “fatty acid supplement*” OR “Omega 3 intake” OR “omega 3 consum*” OR “omega 3 absor*” OR “omega 3 supplement*” OR “Omega 6 intake” OR “omega 6 consum*” OR “omega 6 absor*” OR “omega 6 supplement*” OR “Protein intake” OR “Protein consum*” OR “protein absor*” OR “protein supplement” OR “protein drink” OR “Amino acid intake” OR “amino acid consum*” OR “Amino acid absor*” OR “amino acid supplement*” OR “Carbohydrate intake” OR “carbohydrate consum*” OR “carbohydrate absor*” OR “carbohydrate supplement*” OR “Sugar intake” OR “sugar consum*” OR “sugar absor*” OR “Sucrose intake” OR “sucrose consum*” OR “sucrose absor*” OR “Glucose intake” OR “glucose consum*” OR “glucose absor*” OR “Fructose intake” OR “fructose consum*” OR “Fructose absor*” OR “Fibre intake” OR “fibre consum*” OR “fibre absor*” OR “fibre supplement*” OR “Fiber intake” OR “Fiber

	consum*” OR “fiber absor*” OR “Fiber Supplemet” OR “Starch intake” OR “ starch consum*” OR “starch absor*” OR “Food” OR “Vegetable*” OR “Fruit*” OR “Fast Food” OR “Vegan” OR “Vegetarian” OR “Omnivorous” OR “Carnivorous” OR “Meat” OR “Fish” OR “Legume*” OR “Spice” OR “Spices” OR “Nut” OR “Nuts” OR “Seed*” OR “Whole Grain*” OR “Dairy” OR “Milk” OR “Cheese” OR “Yogurt” OR “Egg” OR “Eggs” OR “Olive” OR “Calorie” OR “Calori* intake” OR “Energy intake” OR “Sweetened beverage” OR “Alcohol” OR “Wine” OR “Caffeine” OR “Coffee” OR “Tea” OR “Water” OR “Soft Drink” OR “Soda” OR “Vitamin intake” OR “vitamin consum*” OR “vitamin absor*” OR “vitamin supplement*” OR “Vitamin drink” OR “Mineral intake” OR “mineral absor*” OR “mineral supplement” OR “mineral drink” OR “Antioxidant*”
Terms “NOT” included	“Acute pain” OR “subacute pain” OR "baby" OR “New Born” OR "Infant" OR “Premature” OR “Postmature“ OR “Infants” OR "P?ediatric" OR "Children" OR "child" OR “Adolescent” OR “Adolescents” OR "preschool" OR “Primary School” OR "Animal study" OR "animal research" OR “animal experiment*” OR "rat" OR "rats" OR "mice" OR "mouse" OR “Hamster” OR “Hamsters” OR “Rabbit” OR “Rabbits”
